# Single and Combined Effects of Deoxynivalenol Mycotoxin and a Microbial Feed Additive on Lymphocyte DNA Damage and Oxidative Stress in Broiler Chickens

**DOI:** 10.1371/journal.pone.0088028

**Published:** 2014-01-31

**Authors:** Wageha A. Awad, Khaled Ghareeb, Agnes Dadak, Michael Hess, Josef Böhm

**Affiliations:** 1 Department for Farm Animals and Veterinary Public Health, Clinic for Avian, Reptile and Fish Medicine, University of Veterinary Medicine, Vienna, Austria; 2 Department of Animal Hygiene, Behaviour and Management, Faculty of Veterinary Medicine, South Valley University, Qena, Egypt; 3 Department for Farm Animals and Veterinary Public Health, Institute of Animal Nutrition and Functional Plant Compounds, University of Veterinary Medicine, Vienna, Austria; 4 Department of Biomedical Sciences, Institute of Pharmacology and Toxicology, University of Veterinary Medicine, Vienna, Austria; Bambino Gesu' Children Hospital, Italy

## Abstract

The immune and intestinal epithelial cells are particularly sensitive to the toxic effects of deoxynivalenol (DON). The aim of this experiment was to study the effects of DON and/or a microbial feed additive on the DNA damage of blood lymphocytes and on the level of thiobarbituric acid reactive substance (TBARS) as an indicator of lipid peroxidation and oxidative stress in broilers. A total of forty 1-d-old broiler chicks were randomly assigned to 1 of 4 dietary treatments (10 birds per group) for 5 wk. The dietary treatments were 1) basal diet; 2) basal diet contaminated with 10 mg DON/kg feed; 3) basal diet contaminated with 10 mg DON/kg feed and supplemented with 2.5 kg/ton of feed of Mycofix Select; 4) basal diet supplemented with Mycofix Select (2.5 kg/ton of feed). At the end of the feeding trial, blood were collected for measuring the level of lymphocyte DNA damage of blood and the TBARS level was measured in plasma, heart, kidney, duodenum and jejunum. The dietary exposure of DON caused a significant increase (P = 0.001) of DNA damage in blood lymphocytes (31.99±0.89%) as indicated in the tail of comet assay. Interestingly addition of Mycofix Select to DON contaminated diet decreased (P = 0.001) the DNA damage (19.82±1.75%) induced by DON. In order to clarify the involvement of lipid peroxidation in the DNA damage of DON, TBARS levels was measured. A significant increase (P = 0.001) in the level of TBARS (23±2 nmol/mg) was observed in the jejunal tissue suggesting that the lipid peroxidation might be involved in the DNA damage. The results indicate that DON is cytotoxic and genotoxic to the chicken intestinal and immune cells and the feed additive have potential ability to prevent DNA damage induced by DON.

## Introduction

Deoxynivalenol (DON), a common contaminant of poultry feed, is mainly produced by *Fusarium graminearum* and *F. Culmorum* and frequently detected in cereals and grains, particularly in wheat, barley, maize and their by-products. Because mycotoxin production depends on environmental conditions such as temperature and humidity, DON contamination can not be avoided completely. Consequently, exposure of human and farm animals including poultry to DON is a permanent health risk assessment issue.

Deoxynivalenol-induced oxidative stress and mitogen-activated protein kinase activation leads to a phenomenon known as ribotoxic stress response, where DON binds to ribosomes and inhibits protein synthesis [1]. It is known that the tissues with a high protein turnover such as the gastrointestinal tract and immune cells are highly sensitive to DON. One of the possible adverse impacts of mycotoxin in cells is the higher production of free radicals and reactive oxygen species leading to oxidative damage [2]. For example, it is assumed that DON and T-2 toxin are capable to increase the free radical production resulting in membrane and DNA damage, consequently oxidative stress is significant mechanism for their toxicity [3]–[8]. DON at concentrations of 3.75–15 µM caused an increase in the levels of TBARS as an index of cellular lipid peroxidation in a dose-dependent manner in HepG2 cells and caused DNA damage [9]. However, the effects of DON on lipid peroxidation are poorly studied in poultry.

Lipid peroxidation can be one indicator for cellular damage in the toxicity of DON mycotoxin. The biomolecules such as nucleic acids, proteins, and lipids are the main targets of oxidative damage [10]. It was shown that trichothecences induced lipid peroxidation and consequently affected cellular membrane integrity and induced metabolic disturbances in animals [6]. Moreover, it was suggested that oxidative stress induced by DON could be one mechanism causing DNA damage in the liver of rat [11]. These effects of DON are rarely investigated in poultry, only a few studies indicating that DON induces DNA damage. For example, 10 mg DON/kg feed produced DNA fragmentation in spleen leukocytes of male broiler chickens [12]. The same dose of DON with the contribution of low protein content was also capable of producing DNA damage of the circulating lymphocytes of male broilers [13]. Even though oxidative stress parameters were not affected in the previous experiments [12], [13], oxidative stress cannot be ignored as a mechanism of DNA damage in DON toxicity [12].

Furthermore, dietary supplement could offer a solution to overcome the genotoxicity of DON on lymphocytes. Dietary nucleotides are nutrients required in certain pathological condition that need intensive nucleic acid and protein synthesis [12]. It was shown that supplementation of broiler diet with dietary nucleotide was beneficial to reverse the genotoxicity of T-toxin on the percentage of DNA damage of spleen leucocytes of chickens also found [12]. A similar trend was found in case of DON feeding (10 mg DON/kg feed), however, the counteracting effect of nucleotide inclusion did not reach significance [12]. Alternatively, dietary addition of microbial supplement seems to be a promising solution to counteract the toxic effects of DON on the percentage of DNA damage and oxidative stress. Recently, a microbial based feed additive was reported to have the ability to reduce the negative impacts of DON in broilers such as the genotoxic and immunotoxic effects [13], [14]. Indeed, supplementation of DON contaminated feed (10 mg/kg feed) with microbial supplement was able to reverse the impacts of DON on DNA damage of blood lymphocytes [13], lipid and protein metabolism, heterophil∶lymphocyte ratio (stress index) and on the antibody immune response to infectious bronchitis virus vaccine [14]. However, these effects of DON and a microbial feed supplement were demonstrated with the contribution of low protein content of broiler feed.

Therefore, the current study was conducted to investigate the effects of chronic oral exposure of DON on circulating lymphocyte DNA damage and on the levels of TBARS in plasma, and other internal organs including intestines in broiler chickens. Additionally, the ability of a microbial based feed additive to counteract the toxic effects produced after chronic DON exposure was also investigated.

## Materials and Methods

### Ethics statement

The animal experiments were discussed and approved by the institutional ethics committee of the University of Veterinary Medicine. All husbandry practices and euthanasia were performed with full consideration of animal welfare.

### Birds and housing

Male broiler chicks (Ross 308) were reared in battery cages from 1-d-old to 35-d-old. The climatic conditions and lighting program were computer-operated and followed the commercial recommendations. Environmental temperature in the first week of life was 35°C and reduced to 25°C until the end of the experiment. During the first week, 22 h of light was provided with a reduction to 20 h afterward.

### Diets

Birds of each group (n = 10) received one of the following dietary treatments; 1) basal diet (control group); 2) basal diet artificially contaminated with pure DON in the dose of 10 mg DON/kg feed (DON group); 3) basal diet artificially contaminated with pure DON in the dose of 10 mg DON/kg feed and supplemented with a microbial feed additive, Mycofix® Select (Biomin GmbH, Herzogenburg, Austria) (2.5 kg/ton of feed) (DON+Mycofix group); 4) basal diet supplemented with a microbial feed additive, Mycofix Select (2.5 kg/ton of feed) (Mycofix group). The birds were fed the starter feed from 1–13 days old and grower feed from 14–35 days old. Composition and analysis of the starter and grower diets are presented in Table 1. Water and feed were available *ad libitum*. Water and feed (in a mash form) were provided in small plastic drinkers and feeders inside the battery cages of each group during the first week of experiment. Afterwards, one long metal drinker and one long metal feeder were connected to each wire frame battery cage instead of plastic drinkers and feeders.

**Table 1:** Composition and analysis of the experimental diet (%).

Ingredient	Starter	Grower
**Corn**	55.0	62.0
**Soya high protein**	29.0	23.80
**Rapeseed oil**	1.00	2.00
**Soybean roasted**	6.91	5.53
**Calcium carbonate**	2.03	1.62
**Monocalcium phosphate**	1.89	1.71
**Palm oil**	1.88	1.50
**Pumpkin seed expeller** 1	0.72	0.58
**Sodium chloride**	0.48	0.38
**L-Lysine HCL 98%**	0.34	0.27
**DL-Methionine 99.5%**	0.24	0.20
**Vitamin Premix** 2	0.16	0.13
**Magnesium phosphate**	0.13	0.10
**L-Threonine**	0.12	0.10
**Trace element premix** 3	0.10	0.08
**Calculated composition**		
**DM**	89	89
**CP**	21.5	18.8
**ME (MJ/kg)**	12.6	13
**Crude fiber**	2.6	2.6
**Crude fat**	6.5	7.2
**Lys**	1.44	1.21
**Met**	0.56	0.48
**Ca (g/kg)**	1.2	1
**P (g/kg)**	0.86	0.72
**Na (g/kg)**	0.2	0.16
**Mg (g/kg)**	0.18	0.17
**Analyzed composition**		
**CP**	23.9	20.9
**Crude fat**	7.0	7.6
**Ca (g/kg)**	14.9	11.2
**P (g/kg)**	8.7	8.7
**Na (g/kg)**	2.85	1.98
**Mg (g/kg)**	3.2	2.5

1 Pumpkin seed expeller is a byproduct of oil manufacture, obtained by pressing of pumpkin seeds, Cucurbita maxima Duch, moschata (Duch) Poir., Cucurbita pepo L., and other species of Cucurbita.

2 Produced by MIAVIT GmbH & Co. KG, Essen (Oldb.), Germany. Each kilogram of vitamin premix contains vitamin A, 200,000 IU; vitamin D3, 80,000 IU; vitamin E, 1,600 mg; vitamin K3, 34 mg; vitamin C, 1,300 mg; vitamin B1, 35 mg; vitamin B2, 135 mg; vitamin B6, 100 mg; vitamin B12, 670 µg; nicotinic acid, 1,340 mg; calcium pantothenic acid, 235 mg; choline chloride, 8,400 mg; folic acid, 34 mg; biotin, 3,350 µg; and methionine, 30 g.

3 Produced by MIAVIT GmbH & Co. KG, Essen (Oldb.), Germany. Each kilogram of trace element premix contains calcium, 196 g; phosphorous, 64 g; sodium, 30 g; magnesium, 6 g; copper, 400 mg; zinc, 1,200 mg; iron, 2,000 mg; manganese, 1,200 mg; cobalt, 20 mg; iodine, 40 mg; selenium, 8 mg.

### Absolute and relative organs weights and sampling

At the end of the feeding trial (35-d-old), all birds were slaughtered after manual stunning with restraint and all organs were rapidly collected and cooled with crashed ice. The weights of gizzard, pancreas, heart, liver, spleen, kidney, bursa, thymus, lung, and brain were recorded. Consequently, the weight of each organ relative to body weight was calculated. Parts of duodenum, jejeunum, heart, and kidney were collected and frozen at −80°C to evaluate the TBARS. During slaughtering, blood was collected in heparinised tubes (with Lithium Heparin as anticoagulant) and plasma was separated by centrifugation at 1000 g for 15 min and stored at −80°C until the time of analysis of TBARS concentration. Moreover, directly before slaughtering, 4 ml of fresh blood was collected from the brachial wing vein of each individual chick in EDTA tubes using standard blood collection procedures for measuring the level of DNA damage of the circulating lymphocytes. One person hold the bird horizontally on its back, one hand collected the legs and the other hand supported the back. Another person pulled the wing of the bird and collected fresh blood from the wing vein. Blood samples were transported immediately in an ice box to the laboratory for lymphocyte isolation. Lymphocytes were separated from the whole blood using a Ficoll lymphocyte isolation medium (Sigma, St. Louis, MO), carefully aspirated by a Pasteur pipette and washed twice with phosphate buffered saline (PBS). Then the lymphocytes were suspended in PBS for measuring the DNA damage using Comet assay. The viability of lymphocytes was counted by trypan blue differential counting with a hemocytometer, and the concentration of cells was adjusted to 1,500 cells/1 µL of PBS.

### Thiobarbituric acid reactive substance (TBARS) estimation

Thiobarbituric acid reactive substances (TBARS) as an index for lipid peroxidation and oxidative stress were estimated in the plasma, kidney, heart, duodenum and jejunum according to the method described earlier [15].

### Blood Lymphocyte DNA damage (Comet Assay)

At 35-d-old, blood was collected and the degree of lymphocyte DNA damage was measured by comet assay (BioCat GmbH, Heidelberg, Germany). The comet assay for cytotoxicity testing was performed according to Steenkamp et al. [16] with some changes. An appropriate cell suspension (40 µL) of isolated lymphocytes was diluted in 400 µL of 0.5% low-melting point argarose and 100 µL were pipette onto glass slides precoated with 1% high-melting point agarose. After lysis (performed in prechilled lysis solution), slides were placed in a comet assay tank (Trevigen Inc., Gaithersburg, MD) and incubated in prechilled electrophoresis buffer. Electrophoresis was performed at 25 V, 300 mA for 20 min in the comet assay tank (chilled with ice). Afterward, slides were washed in neutralization buffer and dH_2_O, fixed in 80% ethanol, and stained according to the instructions of the comet assay silver staining kit (Trevigen Inc.). Three slides were prepared for each sample. Fifty randomly chosen cells were scored per slide (total 150 cells). Comet parameters were analyzed by CometScore (TriTek Corp., Sumerduck, VA).

### Statistical Analysis

The statistical program SPSS (version 17; SPSS GmbH, SPSS Inc., Munich, Germany) was used for data analysis. The Kolmogorov-Smirnov test was used to test the normal distribution of the data. An ANOVA was performed between the 4 groups, followed by Duncan test to find the significance between dietary treatments. The probability values of 0.05 (P≤0.05) were considered significant.

## Results

### Organs weights

The absolute weight of kidney showed a significance difference between control group and birds fed DON contaminated diet either with or without Mycofix Select addition (Table 2).

**Table 2:** Effects of deoxynivalenol and a microbial feed additive on the absolute weights of organs in broiler chickens.

Organ	Control group	DON group	DON+Mycofix group	Mycofix group	P value
**Gizzard (g)**	29.79±4.48	32.22±2.77	32.12±4.39	29.95±4.36	0.520
**Pancreas (g)**	4.57±0.30	4.39±0.34	4.37±0.29	4.23±0.34	0.902
**Heart (g)**	9.86±0.62	9.21±0.43	9.74±0.37	9.39±44	0.747
**Spleen (g)**	1.86±0.10	1.86±0.21	1.73±0.12	1.78±0.16	0.911
**Kidney (g)**	11.33^a^±0.73	9.66^b^±0.44	10.01^b^±0.25	10.77^a^±0.59	0.059
**Liver (g)**	35.64±1.71	34.85±1.81	36.01±1.99	36.33±2.29	0.965
**Lung (g)**	14.99±1.64	11.43±0.92	13.09±1.16	13.51±1.41	0.281
**Thymus (g)**	11.44±1.42	11.30±0.88	11.31±0.54	11.87±0.87	0.970
**Brain (g)**	2.75±0.05	2.81±0.07	2.89±0.03	3.06±0.24	0.376
**Bursa (g)**	3.69±0.37	4.39±0.38	4.14±0.34	4.11±0.22	0.545

a,b Means within the same row with different superscripts are significantly different (ANOVA followed by Duncan test; n = 10/treatment).

The relative weight of kidney showed a reduction (P<0.1) for DON fed birds, however, the relative weight of gizzard increased (P<0.1) (Table 3). Additionally, addition of Mycofix Select to DON contaminated diet counteracted (P<0.1) the effects of DON on relative weight of gizzard.

**Table 3:** Effects of deoxynivalenol and a microbial feed additive on the relative weights of organs in broiler chickens.

Organ	Control group	DON group	DON+Mycofix group	Mycofixgroup	P value
**Gizzard %**	1.63^b^±0.08	1.86^a^±0.05	1.68^b^±0.07	1.65^b^±0.08	0.057
**Pancreas %**	0.25±0.01	0.25±0.01	0.24±0.01	0.23±0.01	0.589
**Heart %**	0.54±0.02	0.53±0.02	0.55±0.02	0.52±0.02	0.856
**Spleen %**	0.10±0.01	0.11±0.01	0.10±0.01	0.10±0.01	0.774
**Kidney %**	0.61^a^±0.02	0.55^b^±0.01	0.56^ab^±0.01	0.59^ab^±0.02	0.071
**Liver %**	1.94±0.05	1.99±0.08	2.01±0.10	1.99±0.08	0.950
**Lung %**	0.78±0.07	0.64±0.04	0.71±0.04	0.68±0.06	0.307
**Thymus %**	0.60±0.05	0.65±0.04	0.63±0.02	0.66±0.04	0.704
**Brain %**	0.15±0.01	0.16±0.01	0.16±0.01	0.17±0.01	0.500
**Bursa %**	0.21±0.02	0.26±0.02	0.23±0.02	0.23±0.02	0.474

a,b Means within the same row with different superscripts are significantly different (ANOVA followed by Duncan test; n = 10/treatment).

### Thiobarbituric acid reactive substance (TBARS)

TBARS levels in plasma, Heart, kidney and duodenum did not show significant changes (P>0.05) due to DON exposure (Table 4). However, TBARS level of jejunal tissue was increased (P<0.001) after chronic exposure to DON, suggesting that jejunum is the target tissue for DON toxicity. Furthermore, Mycofix addition to DON contaminated feed did not have effect on reducing TBARS level of the jejunal tissue (Table 4).

**Table 4:** Effects of dietary DON and a microbial feed additive on thiobarbituric acid reactive substance (TBARS) as an indicator of oxidative stress.

TBARS	Control group	DON group	DON+Mycofix group	Mycofix group	P value
**Plasma (nmol/ml)**	280±48	205±23	239±62	211±27	0.618
**Heart (nmol/mg)**	35±1	30±2	36±2	33±3	0.241
**Kidney (nmol/mg)**	42±3	43±3	42±3	40±4	0.952
**Duodenum (nmol/mg)**	30±5	30±5	26±6	27±6	0.930
**Jejunum (nmol/mg)**	19^b^±1	23^a^±2	23^a^±3	18^b^±2	0.001

a,b Means within the same row with different superscripts are significantly different (ANOVA followed by Duncan test; n = 10/treatment).

### Lymphocyte DNA damage

**Figure 1:**
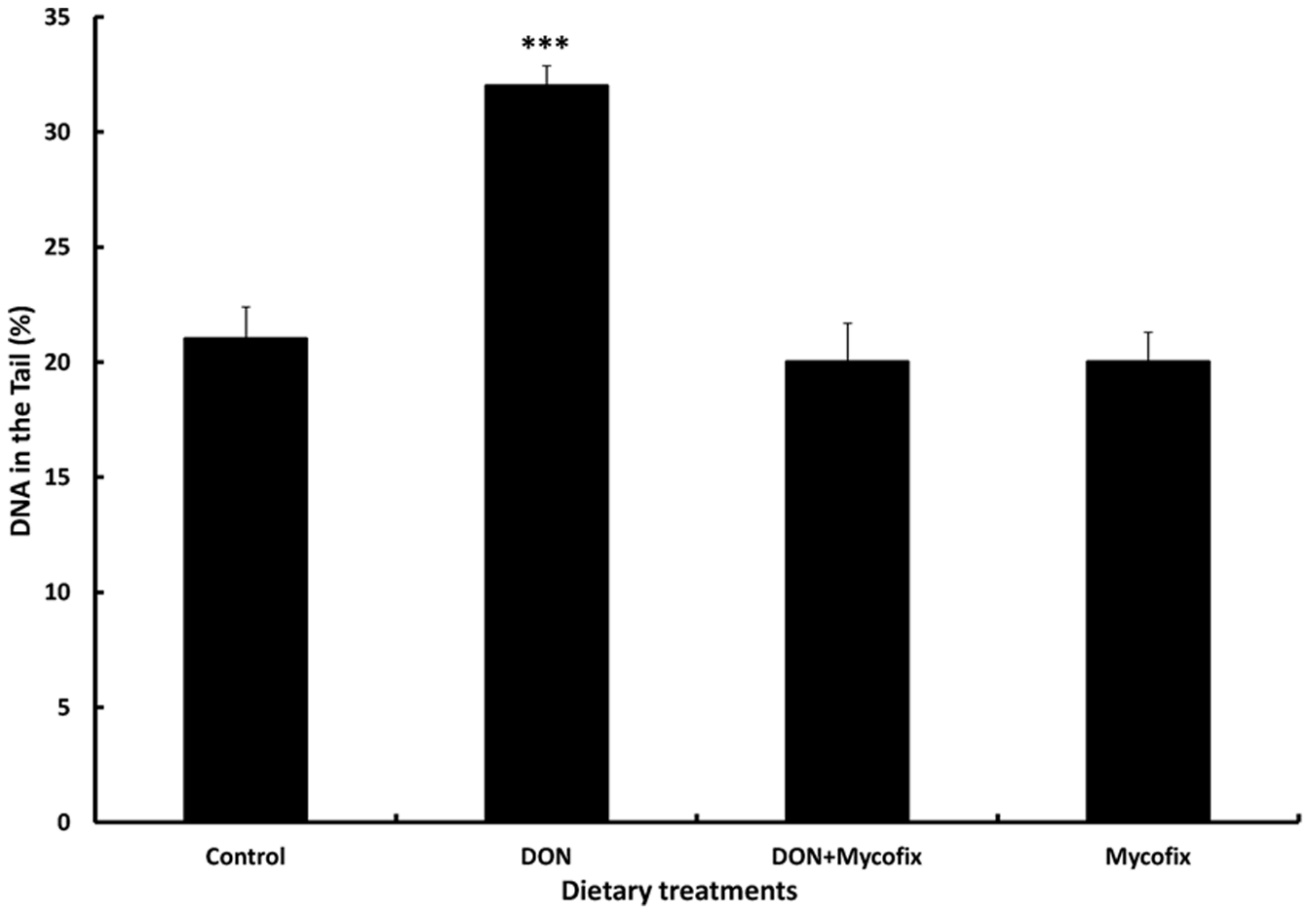
Effect of DON and a microbial feed additive on the blood lymphocyte DNA damage. DNA damages were estimated by Comet assay. Bars show the means and standard errors of means (SEM). Values are the means of 10 birds per treatment. The differences between birds was assessed by ANOVA followed by Duncan test and comparisons were significantly different at P<0.001 (***).

Feeding of DON for 5 weeks increased (P<0.001) significantly the % of DNA damage of blood lymphocytes (Figure 1). However, the addition of Mycofix Select to broiler diet contaminated with DON reduced (P<0.001) the adverse effects of DON on % of DNA damage of blood lymphocytes to a % of damage which is comparable to that of the control group (Figure 1).

## Discussion

Oral exposure of deoxynivalenol was shown to alter the structure and function of chicken intestines as a consequence for the effects of DON on the protein synthesis and nutrient transporters expression [17]–[23]. However, the possibility of DON to induce lipid peroxidation, oxidative stress and genotoxicity was not extensively studied in poultry. Therefore, the current study was conducted to investigate the impact of DON on the oxidative stress by measuring the level of TBARS in the intestine and internal organs and to estimate the DNA damage induced by DON in broiler chickens. Additionally, the efficacy of a microbial based feed additive to counteract or reduce DON impacts was also investigated.

The toxicity of DON is occurred by different mechanisms of action. DON binds to the eukaryotic 60 S ribosomal subunit causing inhibition of protein, DNA and RNA synthesis [24]. Moreover, it was shown that DON stimulated lipid peroxidation and induced DNA damage [7], [9], [11]. In the present study DON increased the TBARS level in the jejunal tissue of broilers, suggesting that DON produced lipid peroxidation and oxidative stresss in the jejunal cells. It was known that DON produces free radicals that induce lipid peroxidation, leading to alteration of membrane integrity, cellular redox signaling and changes in the cell antioxidant status [25]. Furthermore, it was showed that DON had negative impacts on the morphology and functionality of the jejunal villi [17], [18].

Interestingly, exposure of the cells to DON caused a significant increase of DNA migration in comet assay after DON addition in the dose of 3.75–30 µM, indicating that DON caused DNA strand breaks [9]. The involvement of lipid peroxidation in the DNA damage induced by DON was established by increasing levels of thiobarbituric acid-reactive substances (TBARS), suggesting that the DNA damage induced by DON in HepG2 cells is attributed possibly to the oxidative stress [9]. However, *in vivo* studies in poultry failed to document the association between lipid peroxidation induced by DON feeding and the genotoxic effects of DON [12], [13]. As the genotoxicity of DON was not widely established, the exact mechanistic action of DON to cause DNA damage remains unclear. DON may cause direct DNA brakes or acts by different epigenetic mechanisms such as DNA base oxidation and methylation which may alter the gene expression and cell signalling and thus inhibit proliferation and cause cell death. The obtained DNA fragmentations in the Comet assay, are a result of excision and repair of oxidised bases [12]. In the current study, DON induced DNA fragmentation of blood lymphocytes of broiler chickens measured by Comet assay and existing as % of DNA in the tail of the comet. Similarly, feeding of chickens with 10 mg DON/kg feed produced DNA damage of spleen leucocytes [12]. Recently, the genotoxic effect of DON with the contribution of low protein content of broiler feed was reported [13] without production of lipid peroxidation and oxidative stress in the plasma and liver. The results of the current study showed also that DON did not affect the level of TBRAS in the plasma, heart, kidney and duodenum of broiler chickens. Similarly, the dietary contamination with DON at the level 3 mg/kg did not affect malondialdehyde (MDA) contents in duodenal mucosal tissue of broilers [26]. However, DON increased the TBARS level in jejunal tissue in the current study, indicating that the jejunal mucosa is more sensitive for the oxidative stress produced by DON toxicity. It was found that diet contaminated with DON induced oxidative stress and compromised the blood phagocytic activity in fattening chickens [27]. DON increased the production of MDA, a biomarker of lipid peroxidation in human intestinal Caco-2 cells [28]. Thus, it can not be excluded that oxidative pathways may be one of the mechanisms by which DON induces DNA damage.

It is known that the relative organ weight could be affected after ingestion of mycotoxins. Therefore, it can be expected that the relative weights of spleen and bursa of Fabricius might be affected after DON intoxication, since the later can cause malfunction of immune organs. This effect was reported in chickens fed DON [29]. However, in the current study the relative weights of both gizzard and kidney are affected after oral DON exposure which could be a consequence of irritation and intensive function of these organs. Similar effect was reported in chickens fed 10 mg DON/kg feed [12], which resulted in higher relative weight of gizzard. Furthermore, both absolute and relative weight of kidney was decreased after 5 weeks of DON feeding which could indicate that DON damaged the kidney cells. In contrast to this finding, DON was shown to increase the relative weight of kidney [12]. This alteration in gizzard was removed when Mycofix Select was added to the DON contaminated feed. However, the alteration in the relative weight of kidney was not removed when Mycofix Select was supplemented to DON contaminated feed. Moreover, Mycofix Select alone did not change the absolute and relative weight of gizzard and the relative weight of kidney indicating that these organs remains unaffected by dietary addition of this microbial supplement. It is known that the relative weight of organ is an indicator for the toxicity rather than the absolute weight of organ. In this context, the slight reduction of the absolute weight of kidney after 5 weeks of Mycofix Select addition to non contaminated feed did not lead to conclude an adverse effect of microbial supplement because the relative weight of kidney did not change. Furthermore, the reduction of the relative weight of kidney and the higher relative weight of gizzard after DON intoxication could suggest that DON induced a toxicological impact that may include either atrophy or enlargement of the target organ, probably due to cell damage or irritation respectively.

Mycofix Select is a microbial feed additive and capable to cope with DON [30]. In this experiment, addition of Mycofix Select to DON contaminated diet was beneficial to reduce the risk of DON in the terms of decreasing the DNA damage of blood lymphocytes caused by DON. However, Mycofix Select could not fix the negative effects of DON on TBARS of jejunal tissue. The ability of this microbial feed additive to reverse DON induced DNA damage of circulating lymphocytes but not DON induced oxidative stress in jejunum could be related to the dose of Mycofix Select added to DON contaminated diet. In the current experiment, Mycofix Select was added in the dose of 2.5 kg per ton of contaminated feed which could not be sufficient to reverse all the negative effects of 10 mg DON/kg feed.

In conclusion, from the current experiment it can be concluded that feeding of broilers with deoxynivalenol contaminated feed for 5 weeks resulted in a significant health risk such as lipidperoxidation and oxidative stress of jejunal cells and damaging the DNA of blood lymphocytes. The results indicate also that DNA damage induced by DON is probably through oxidative stress. In addition, the microbial feed additive (Mycofix Select) can reduce the risk of DNA damage in immune cells caused by DON, which underlines their possible beneficial effect on the immune system in DON intoxication.
