# Correction: Single and Combined Effects of Deoxynivalenol Mycotoxin and a Microbial Feed Additive on Lymphocyte DNA Damage and Oxidative Stress in Broiler Chickens

**DOI:** 10.1371/journal.pone.0100907

**Published:** 2014-06-16

**Authors:** The PLOS ONE Staff

The affiliation for the fifth author is incorrect. Josef Böhm is not affiliated with #2 but with #3, Department for Farm Animals and Veterinary Public Health, Institute of Animal Nutrition and Functional Plant Compounds, University of Veterinary Medicine, Vienna, Austria
